# Dyslipidemia rather than Type 2 Diabetes Mellitus or Chronic Periodontitis Affects the Systemic Expression of Pro- and Anti-Inflammatory Genes

**DOI:** 10.1155/2017/1491405

**Published:** 2017-02-20

**Authors:** Rafael Nepomuceno, Bárbara Scoralick Villela, Sâmia Cruz Tfaile Corbi, Alliny De Souza Bastos, Raquel Alves Dos Santos, Catarina Satie Takahashi, Silvana Regina Perez Orrico, Raquel Mantuaneli Scarel-Caminaga

**Affiliations:** ^1^Department of Morphology, School of Dentistry at Araraquara, São Paulo State University (UNESP), 14801-903 Araraquara, SP, Brazil; ^2^Department of Diagnosis and Surgery, School of Dentistry at Araraquara, São Paulo State University (UNESP), 14801-903 Araraquara, SP, Brazil; ^3^Postgraduate Program in Sciences of the University of Franca, 14404-600 Franca, SP, Brazil; ^4^Department of Genetics, Faculty of Medicine of Ribeirão Preto, University of São Paulo (USP), 14040-900 Ribeirão Preto, SP, Brazil; ^5^Department of Biology, Faculty of Philosophy, Sciences and Letters at Ribeirão Preto, University of São Paulo (USP), 14040-900 Ribeirão Preto, SP, Brazil

## Abstract

A high percentage of type 2 diabetes mellitus (T2D) patients are also affected by dyslipidemia and chronic periodontitis (CP), but no studies have determined the gene expression in patients that are simultaneously affected by all three diseases. We investigated the systemic expression of immune-related genes in T2D, dyslipidemia, and CP patients. One hundred and fifty patients were separated into five groups containing 30 individuals each: (G1) poorly controlled T2D with dyslipidemia and CP; (G2) well-controlled T2D with dyslipidemia and CP; (G3) normoglycemic individuals with dyslipidemia and CP; (G4) healthy individuals with CP; (G5) systemic and periodontally healthy individuals. Blood analyses of lipid and glycemic profiles were carried out. The expression of genes, including* IL10, JAK1, STAT3, SOCS3, IP10, ICAM1, IFNA, IFNG, STAT1, *and* IRF1, *was investigated by RT-qPCR. Patients with dyslipidemia demonstrated statistically higher expression of the* IL10* and* IFNA* genes, while* IFNG, IP10, IRF1, JAK1, *and* STAT3 *were lower in comparison with nondyslipidemic patients. Anti-inflammatory genes, such as* IL10*, positively correlated with parameters of glucose, lipid, and periodontal profiles, while proinflammatory genes, such as* IFNG*, were negatively correlated with these parameters. We conclude that dyslipidemia appears to be the primary disease that is associated with gene expression of immune-related genes, while parameters of T2D and CP were correlated with the expression of these important immune genes.

## 1. Introduction

Type 2 diabetes mellitus (T2D) encompasses individuals who have insulin resistance and who usually have relative (rather than absolute) insulin deficiency and decreased *β*-cell function [1, 2]. T2D is characterized not only by changes in carbohydrate metabolism, but also by changes in the metabolism of lipids, which is defined in the literature as diabetic dyslipidemia. Therefore, it is important to emphasize the fact that T2D may exist concurrently and/or synergistically with other systemic diseases, such as dyslipidemia, since there is a direct relationship between indices of glycemic control and plasma lipid elevation [3, 4]. Moreover, it is estimated that patients with diabetes have a two- to fourfold higher risk of ischemic disease. Dyslipidemia is a major risk factor for macrovascular complications in T2D patients [5]. In patients with diabetes, alteration in the distribution of lipids increases the risk of atherosclerosis [6]. Dyslipidemia in association with diabetes mellitus is the major cause of morbidity and mortality due to the high rate of severe cardiovascular diseases [3].

Dyslipidemia is a very common metabolic abnormality, which is characterized by a spectrum of quantitative and qualitative changes in serum lipids and lipoproteins [7]. The main characteristics of dyslipidemia include high total cholesterol (TC), high triglycerides (TG), elevated low-density lipoprotein (LDL), and decreased high-density lipoprotein (HDL) [8]. The cause of dyslipidemia may be genetic, environmental, or both.

Chronic periodontitis (CP) is an infectious disease that affects dental supporting tissues (attachment and bone loss) and is caused predominantly by Gram-negative anaerobic bacteria [9, 10]. CP induces local and systemic elevation of proinflammatory cytokines, and these molecules contribute to soft and hard periodontal tissue destruction and loss of dental elements, in addition to the prevalence and severity of systemic diseases, such as diabetes and dyslipidemia [11–15]. Reports have suggested that CP and dyslipidemia/diabetes may be involved in a two-way relationship [13–15]. CP has been recognized as the sixth most common complication associated with T2D [16]. Also, T2D patients demonstrated a greater extent and severity of CP compared to normoglycemic individuals [17–19]. Moreover, in recent years, several papers have investigated the relationships between periodontitis and lipid parameters. Dyslipidemia increases the risk for CP, and periodontal inflammation negatively affects serum lipid control [13, 20].

The* IL10, IL10RA, IL10RB, JAK1, STAT3, SOCS3, IP10, ICAM1, IFNA, IFNARA, IFNAR2, IFNG, IFNGR1, IFNGR2, STAT1, *and* IRF1 *genes are important molecules associated with the host immunoinflammatory response. There are limited studies on the association between these genes and the multifactorial diseases T2D, dyslipidemia, and chronic periodontitis. Moreover, there are no studies investigating the expression of these genes in subjects concomitantly affected by these three pathologies. In view of the literature, and because it has been increasingly common to find T2D patients that are also affected by a combination of dyslipidemia and chronic periodontitis, our hypothesis was that glycemic control, lipid profile, and periodontal status can alter the systemic expression of pro- and anti-inflammatory genes. Therefore, the aim of the present study was to evaluate the expression of important genes of the IL10 and interferon-alpha and -gamma pathways in poorly or well-controlled T2D patients and in normoglycemic individuals, both conditions associated with dyslipidemia and chronic periodontitis. In addition, correlation analyses were made in order to determine the influence of the glycemic/lipid levels and periodontal parameters on the expression of these important genes.

## 2. Materials and Methods

### 2.1. Selection Criteria

The study was approved by the Ethics in Human Research Committee of the Araraquara School of Dentistry (UNESP, Univ. Estadual Paulista, Araraquara, Brazil; Protocol number 50/06) and was conducted according to the ethical principles of the Declaration of Helsinki between 2009 and 2011. All volunteers were informed about the aims and methods of this study, and they provided their written consent to participate.

We evaluated 1788 patients, age ranging from 35 to 60 years, similar socioeconomic level and with at least 15 natural teeth. Patients were excluded according to the criteria described in Bastos et al. (2012) [21] and Corbi et al. (2014) [22].

### 2.2. Clinical Record and Physical Evaluation

The individuals answered a structured questionnaire about demographic characteristics, personal and family medical history, and use of medications. A trained examiner collected information regarding time since the diagnosis of diabetes, use of hypoglycemic medication, and presence of diabetes-associated complications. Subjects completed a physical examination including measurement of waist and hip circumference (centimeters), height (meters), weight (kilograms), and body mass index (BMI).

### 2.3. Laboratory Measurements of Metabolic Control and Lipoprotein Profile

The same laboratory performed all analyses, and blood samples were collected after a 12 h overnight fast for the evaluation of fasting plasma glucose (mg/dL) by a modified Bondar and Mead method [23]. Glycated hemoglobin (HbA1c) was measured by enzymatic immunoturbidimetry, insulin levels were measured by the chemiluminescence method (U/L), and lipid profile (TC, TG, and HDL) was measured by enzymatic methods. LDL was determined by the Friedewald formula.

Patients were considered as nondiabetics (normoglycemic individuals) if they presented fasting glucose levels <100 mg/dL and HbA1c <6.5%. T2D patients that were diagnosed by an endocrinologist and who monitored their glycemic control by evaluation of HbA1c were divided into poorly controlled patients (HbA1c ≥ 8.5%, 64 mmol/mol) or well-controlled patients (HbAc1 < 7.0%) [24]. Insulin resistance was also evaluated for insulin levels by the chemiluminescence method (*μ*U/mL) for calculation of the homeostasis model assessment (HOMA) of the insulin resistance index [25].

To avoid the inclusion of individuals with transitory dyslipidemia, the cutoff points used were the highest values according to the National Cholesterol Educational Program Adult Treatment III (total cholesterol ≥ 240 mg/dL, LDL ≥ 160 mg/dL, HDL ≤ 40 mg/dL, and TG ≥ 200 mg/dL). Patients were considered affected by dyslipidemia when they presented abnormal levels of at least one of the abovementioned parameters [26].

### 2.4. Periodontal Clinical Examination

All patients were subjected to a periodontal clinical examination performed as described in Corbi et al. (2014) [22]. Chronic periodontitis, as defined by the American Academy of Periodontology [27], includes local signs of inflammation and tissue destruction (presence of deep periodontal pockets ≥ 6 mm) and loss of the connective tissue attachment of gingiva to teeth (clinical attachment loss ≥ 4 mm) in at least four nonadjacent teeth. Severe chronic periodontitis was defined as the presence of deep periodontal pockets ≥ 6 mm with clinical attachment loss of ≥ 5 mm and bleeding on probing in at least eight sites distributed in different quadrants of the dentition (Table 1) [28].

### 2.5. Study Population

After all the examinations described above, 150 patients were selected and divided into five groups (G) containing 30 individuals each based upon diabetic and dyslipidemic status: (G1) poorly controlled diabetics with dyslipidemia and chronic periodontitis; (G2) well-controlled diabetics with dyslipidemia and chronic periodontitis; (G3) normoglycemic individuals with dyslipidemia and chronic periodontitis; (G4) systemically healthy individuals with chronic periodontitis; and (G5) systemically healthy individuals without chronic periodontitis.

Because the patients enrolled here were the same as those investigated in previous studies of our research group, detailed information regarding recruiting, clinical inclusion criteria, and power analysis can be found in Bastos et al. (2012) [21] and Corbi et al. (2014) [22].

### 2.6. Reverse Transcriptase-Real-Time Quantitative PCR (RT-qPCR)

Peripheral venous blood was collected from each subject and immediately centrifuged on a Ficoll-Paque PLUS (GE Healthcare Life Sciences, Oslo, Norway) density gradient, followed by consecutive washings with saline (NaCl 0.9%), to isolate peripheral blood mononuclear cells (PBMCs). Total RNA samples were extracted using TRIzol reagent (Invitrogen, Rockville, MD, USA) according to the manufacturer's instructions. Samples containing total RNA were purified using the RNeasy Protection Mini Kit (Qiagen, Hilden, Germany). RNA was quantified using a NanoVue Spectrophotometer (GE Healthcare Life Sciences, Oslo, Norway), and its integrity was assessed by 1% agarose gel electrophoresis. Absorbances (*λ* = 260/280 and 260/230) were measured, and samples with ratios lower than 1.8 and higher than 2.2 were discarded. cDNA was synthesized using oligo-dT primers and the SuperScript III First-Strand Synthesis Super Mix kit according to the manufacturer's instructions (Invitrogen). Real-time quantification of target mRNA was performed using SYBR Green (Applied Biosystems, Foster City, CA, USA) according to the manufacturer's instructions. Briefly, each amplification was performed in a total volume of 25 *μ*L, containing 12.5 *μ*L SYBR Green Universal PCR Master Mix 2x (Applied Biosystems), 500 nM each forward and reverse PCR primers, and 3 ng of cDNA. Detailed information regarding sequences of primers and real-time PCR cycling parameters are described in previous studies [29, 30] and in Supplemental Table  1 (in Supplementary Material available online at https://doi.org/10.1155/2017/1491405). The investigated target genes were* IL10, IL10RA, IL10RB, JAK1, STAT3, SOCS3, IP10, ICAM1, IFNA, IFNARA, IFNAR2, IFNG, IFNGR1, IFNGR2, STAT1, *and* IRF1*. The specificity of the amplified PCR products was confirmed by dissociation curve analysis. All reactions were performed in duplicate, and the examiner was blinded regarding which group each sample belonged to. Relative quantitation of mRNA was carried out after normalization of the gene expression to the* GAPDH* endogenous control gene. To calculate gene expression, Expression Suite Software (Applied Biosystems) was used, which employs the comparative C*τ*(ΔC*τ*) method for multiple data analysis.

### 2.7. Statistical Analysis

The distribution of the data was evaluated by D'Agostino–Pearson omnibus normality test. Comparisons between all groups for nonnormally distributed variables (e.g., 2^−ΔC*τ*  ^ values of gene expression) were performed by the Kruskal–Wallis test, and Dunn test was used to correct for multiple comparisons. For normally distributed variables, one-way analysis of variance (ANOVA) test with Holm–Sidak multiple comparison test was used. The general characteristics of each group are described with the mean and standard deviation (SD). The significance level was set at *α* = 0.05. GraphPad Prism 5.0 software (GraphPad Inc., USA) was used to perform these statistical analyses. To investigate correlations (adjusted for age and sex) among various studied parameters, Spearman's rank correlation test was performed using SPSS® IBM software version 19. The significance level was set at *α* = 0.05.

## 3. Results

### 3.1. Analysis of the Characteristics of Study Population

From a total of 1788 patients screened, 150 of them (30 patients per group) passed the research inclusion criteria.  Table 2, which is extracted in part from our previous studies [21, 22, 24], showed no significant differences between groups regarding gender. Fasting glucose and HbA1c, as expected, were significantly higher in G1, confirming the poor glycemic control; these parameters were also higher in the G2 group than in nondiabetics (G3, G4, and G5). Insulin levels showed no significant differences between G1 and G2 groups. The HOMA index was significantly higher in the diabetic groups (G1 and G2) than in G3, G4, and G5. BMI, waist-to-hip ratio, and abdominal circumference were higher in diabetic groups (G1 and G2) than in the G5 group. As expected, the total cholesterol, LDL, and triglyceride levels were higher in the G1, G2, and G3 groups than G4 and G5 (Table 2).

Table 1 shows that periodontal tissue destruction (including bone loss) and local inflammation were significantly more severe in diabetics, particularly in the G1 group, which presented a high percentage of periodontal sites with bleeding on probing, probing depth ≥ 6 mm, clinical attachment loss ≥ 5 mm, and suppuration. The G2 group showed significant difference in relation to the presence of deeper periodontal sites when compared to groups without T2D (G3, G4, and G5).

### 3.2. Analysis of Gene Expression

**Figure 1:**
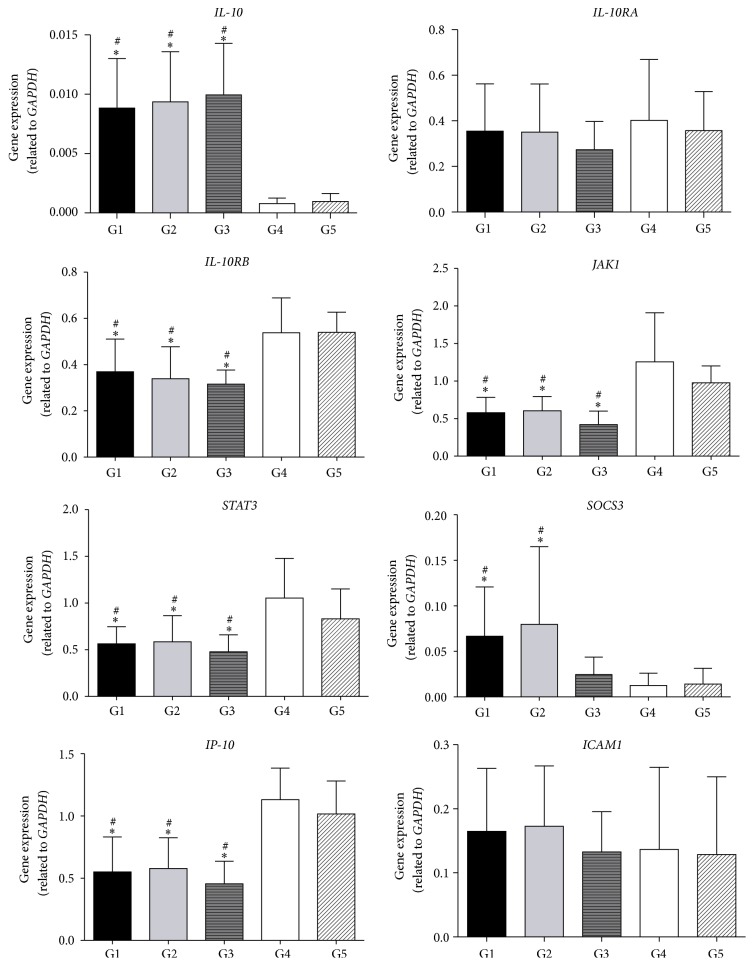
Signaling pathway of* IL10* gene in groups G1, G2, G3, G4, and G5. All values were normalized to* GAPDH*. Data represent the mean ± SD. ^#^*P* ≤ 0.05 compared to G4 group; ^*∗*^*P* ≤ 0.05 compared to group G5. Comparisons between all groups were performed by the Kruskal–Wallis test, and Dunn test was used to correct for multiple comparisons.

**Figure 2:**
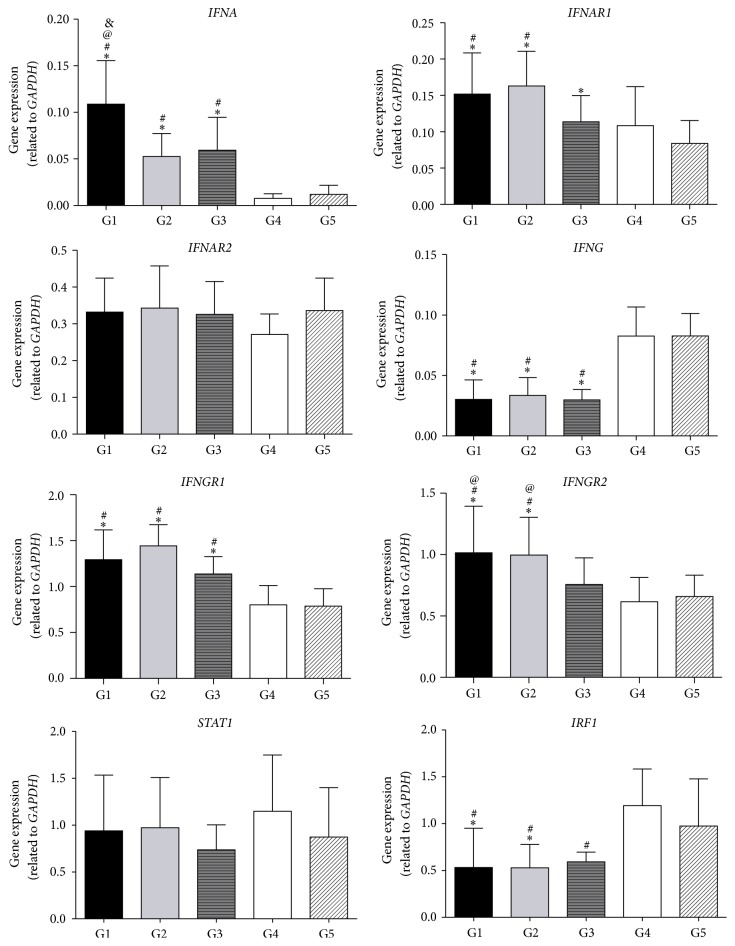
Signaling pathway of* IFNA* and* IFNG* genes in groups G1, G2, G3, G4, and G5. All values were normalized to* GAPDH*. Data represent the mean ± SD. ^&^*P* ≤ 0.05 compared to G2; ^@^*P* ≤ 0.05 compared to G3; ^#^*P* ≤ 0.05 compared to G4; ^*∗*^*P* ≤ 0.05 compared to G5. Comparisons between all groups were performed by the Kruskal–Wallis test, and Dunn test was used to correct for multiple comparisons.

Regarding gene expression analysis, as can be seen in Figure 1 (*IL10* signaling pathway genes) and Figure 2 (*IFNA* and* IFNG* signaling pathway genes),* IL10* and* IFNA *genes were significantly more highly expressed in patients with T2D and dyslipidemia (G1, G2, and G3). The opposite occurred with the* IFNG* gene, which was more highly expressed in the groups without dyslipidemia (G4 and G5) compared to G1, G2, and G3 (Figures 1 and 2). Considering the* IL10* pathway genes,* IL10* was expressed at a significantly higher level in dyslipidemic individuals (G1, G2, and G3) in comparison with G4 and G5, while the* IL10RB, JAK1, STAT3, *and* IP10* genes showed the opposite lower expression (Figure 1). Moreover,* SOCS3 *was expressed at a significantly higher level in T2D patients (G1 and G2). Concerning genes of the interferon-alpha and -beta pathways, expression of the* IFNG, IRF1,* and* JAK1 *genes was significantly lower, while* IFNA* showed higher expression in dyslipidemic individuals in comparison with G4 and G5 groups (Figure 2).

### 3.3. Adjusted Correlation Analysis

Gene expression was significantly correlated with physical parameters and with glycemic and lipid profiles (Table 3). There was a significant positive correlation between* IL10, IFNA,* and* SOCS3* genes with physical, glycemic, and lipid profiles, especially HbA1c (*IFNA*, *r* = 0.65) and total cholesterol (*IL10*, *r* = 0.57). Furthermore, some genes showed independent negative correlation with physical as well as glycemic and lipid profiles, especially triglycerides (*IP10*, *r* = −0.55) and total cholesterol (*IFNG*, *r* = −0.54;* IRF1, r* = −0.49; and* JAK1, r* = −0.44).

Correlation analyses between genes are presented in Table 4. The* IL10* gene correlated negatively with* IP10*,* IRF1, STAT3, JAK1, *and mainly* IFNG *(*r* = −0.75). In addition,* IL10* gene expression positively correlated with* IFNA *and* SOCS3*. The expression of* IFNG* gene was positively correlated mainly with the* IP10* gene (*r* = 0.72) and negatively correlated with* IFNA *(*r* = −0.57). Furthermore,* IRF1* positively and strongly correlated with* STAT3 *and* JAK1.*

Increases in the percentage of the periodontal clinical parameters (visible plaque, marginal bleeding, BP, PPD ≥ 6 mm, NI ≥ 5 mm, and suppuration) indicate the severity of chronic periodontitis [27]. There was a positive correlation between the expression of* IL10* and* IFNA* genes with periodontal clinical parameters, such as visible plaque, marginal bleeding, BP, PPD ≥ 6 mm, CAL ≥ 5 mm, and suppuration. Moreover, there was a negative correlation between* IFNG, IP10, *and* IRF1* genes with those same periodontal clinical parameters. As periodontal pocket depth increased (PPD ≥ 6 mm), the expression of* IL10* and* IFNA* genes increased, while the opposite was observed for the* IFNG* and* IP10* genes (Table 5).

## 4. Discussion

To our knowledge, this is the first study to investigate the expression of genes belonging to the* IL10* and interferon-alpha and -gamma pathways in individuals affected by T2D, dyslipidemia, and CP. Surprisingly, we find difference in gene expression related to poor or good glycemic control of T2D only for the* IFNA* gene. The present results indicate that dyslipidemia seemed to influence systemic expression of important pro- and anti-inflammatory genes in individuals concomitantly affected by the multifactorial diseases of T2D, dyslipidemia, and CP. However, this does not mean that T2D and CP did not influence gene expression, since glucose and periodontal parameters were significantly correlated with expression of the investigated immune genes.

We demonstrated here that dyslipidemic patients had increased expression of the* IL10* gene (Figure 1). In agreement, increased circulating IL-10 levels have been reported in obese [31, 32], T2D [33], coronary heart disease [34], and acute coronary syndrome [35] patients. Increased* IL10 *mRNA levels were shown in osteoblastic cells under high glucose concentration [36]. However, other studies have reported reduced levels of the anti-inflammatory IL-10 in dyslipidemic subjects, together with higher levels of proinflammatory cytokines [32, 37–39]. The mechanisms explaining the paradoxical findings are currently unclear. A possible explanation for our results may be that the increased* IL10* gene expression may represent a delicate regulatory pathway to restore the homeostasis under inflammatory conditions. Thus,* IL10* may be a surrogate marker of a heightened inflammatory process and act to counterregulate the effects of proinflammatory mediators [35, 40]. In regard to the correlation analysis, we demonstrated that* IL10* gene expression was significantly positively correlated with lipid parameters and also with physical and glycemic parameters. Similarly, Bassols et al. (2010) found that serum concentrations of IL-10 had a positive correlation with fasting triglycerides, weight, BMI, waist circumference, and waist-to-hip ratio [31].

*SOCS3* is an important IL10-responsive gene, since SOCS-3 protein inhibits JAK/STAT-dependent signaling by blocking STAT phosphorylation, which inhibits the expression of many proinflammatory genes [41]. IL10 rapidly upregulates transcription of* SOCS3 *[41]. Proinflammatory cytokines may stimulate the production of SOCS-3, which in turn participates in a negative-feedback loop that modulates cytokine action. Both IFN-gamma and IL-10 rapidly induce* SOCS3* gene expression in monocytes [41]. In this study, in agreement with the literature, there was a positive correlation between* SOCS3* and* IL10* genes (Table 4).

Data analysis indicated that T2D patients (G1 and G2 groups) had increased expression of the* SOCS3* gene. Similarly, it was demonstrated that SOCS-3 has been implicated as a mediator of insulin resistance [42], and SOCS-3 protein expression was elevated in skeletal muscle of insulin resistant T2D [43]. It is noteworthy that* SOCS3* expression is increased in mononuclear cells from healthy people in response to a high-fat, high-carbohydrate meal or following ingestion of glucose or cream [44, 45]. Furthermore,* SOCS3 *correlation analyses demonstrated results compatible with those found for* IL10*; that is, significant positive correlations were observed between* SOCS3* expression with glycemic profile and physical and lipid parameters (Table 3).

IFN-alpha has been widely employed in the treatment of various diseases, such as hematologic malignancies and hepatitis B [46, 47]. After beginning IFN-alpha treatment, serum lipids levels increased sharply [46–49]. Indeed, treating patients with this interferon caused marked changes in serum lipoprotein metabolism, leading to dyslipidemia [49]. Therefore, IFN-alpha seems to play an important role in lipid metabolism and accelerated atherosclerosis [50]. Endogenous IFN-alpha has been proposed to play a role in hypertriglyceridemia by its ability to promote hepatic lipogenesis and reduced triglyceride clearance [51]. In agreement, our present results show that* IFNA* expression was higher in groups with dyslipidemia (G1, G2, and G3). Furthermore,* IFNA* expression positively correlated with LDL cholesterol, triglycerides, and total cholesterol (Table 3). Moreover, the expression of* IFNA* was even higher in T2D patients with poor glycemic control (Figure 2), and a strong positive correlation was found between expression of this gene and the percentage of HbA1c. The relationship between* IFNA* gene expression and T2D is presented here for the first time in the literature; further investigation is indicated.

The literature demonstrates that* IFNA* and* IFNG* exert opposing effects on the regulation of cytokine expression, including IL-10 [52, 53]. The enhancement of IL-10 production by IFN-alpha appears to be tightly regulated, since IFN-alpha has a potent effect on the production of IL-10 by activated monocytes and T-helper cells [53]. Here, we found similar results, considering that there was higher expression of* IFNA* and* IL10 *genes in the same groups (Figures 1 and 2), and there was positive correlation between these genes.

In contrast to the* IL10 *and* IFNA* genes, the present study demonstrates lower expression of* IFNG* in the groups composed by patients with dyslipidemia (G1, G2, and G3). There was strong negative correlation between* IL10* and* IFNG*, indicating a lower expression of* IFNG, *accompanied by higher expression of* IL10, *in patients with systemic inflammatory status. IFN-gamma is a major proinflammatory cytokine produced by Th1 cells and natural killer cells and is responsible for macrophage activation and increased production of IL-1 and TNF-alpha [54–56]. Th1 and Th2 cells inhibit each other; for example, IL-10, which is produced by Th2 cells, inhibits Th1 cells, and IFN-gamma inhibits the activation of Th2 [57]. IL10 is a potent inhibitor of the activity of monocytes/macrophages, including oxidative stress and the production of cytokines, such as TNF-alpha, IL-1, and IL-6 [58, 59]. TNF-alpha is an important cofactor for the induction of IFN-gamma by Th1 cells, while the ability of IL-10 to inhibit TNF-alpha production has been one of the mechanisms by which IL-10 inhibits IFN-gamma [60, 61]. Therefore, our results agree with these concepts.

PBMCs containing phytohemagglutinin-stimulated lymphocytes of dyslipidemic patients have been shown to release higher IFN-gamma than controls [62]. Since IFN-gamma has been detected in human atherosclerotic plaques, it is accepted that IFN-gamma is a proatherogenic cytokine, and its action occurs through the activation of macrophages, with subsequent accumulation of lipids [63]. In disagreement with these studies, other studies have demonstrated that (i) severe hypercholesterolemia in mice decreases IFN-gamma response and induces IL-10 response [64]; (ii) PBMCs incubated in high glucose concentrations produced higher levels of TNF-alpha, IL-1beta, and IL-6, while IFN-gamma did not change [65]; (iii) studies with whole blood or PBMCs of T2D patients with and without tuberculosis reported decrease in IFN-gamma production [66–68]. Similarly, we found a negative correlation between the* IFNG* gene with glycemic and lipid profiles; furthermore, the patients with dyslipidemia (G1, G2, and G3) expressed lower* IFNG* mRNA levels than systemically healthy patients (G4 and G5).

IFN-gamma demonstrates synergism with proinflammatory cytokines, such as IL1-beta, and also induces the expression of interferon-gamma-induced protein-10 (IP-10, also called CXCL10) [41, 69, 70]. In this study, the* IP10* gene expression was strongly positively correlated with the* IFNG* gene (*r* = 0.72). IL-10 is able to inhibit the expression of* IP10*, which agrees with the strong negative correlation found between* IP10* and* IL10 *(*r* = −0.67). This occurs because IL-10 and IFN-gamma compete for intracellular mechanisms, and IL-10 causes inhibition of STAT-1 activation in the IFN-gamma signaling pathway [52].

A significant reduction of circulating IP-10 concentration was observed as a consequence of triglyceride ingestion [71]. In our study,* IP10* gene expression negatively correlated with T2D and dyslipidemia parameters. In addition,* IP10* gene expression was significantly lower in patients with dyslipidemia (G1, G2, and G3 groups).

Similar to* IP10*, intercellular adhesion molecule 1 (*ICAM1*) is also stimulated by IFN-gamma, while* IL10* can inhibit its expression [41, 70]. ICAM1 is an important inflammatory marker, which was associated with metabolic syndrome by multiple linear regression models [72]. However, the results obtained here demonstrate that the ICAM 1 gene was not differentially expressed between the five groups.

Interferon regulatory factor 1 (IRF1) is an important signaling protein in the interferon pathway [73, 74], in which IFN-gamma is able to activate its transcription [75]. In the same way of* IFNG, IRF1* was higher in patients without dyslipidemia. Concerning the* STAT1* gene, it is under direct transcriptional regulation by IFNs, but it may also be regulated by* IRF1* positive-feedback mechanisms, leading to an increase in* STAT1* gene expression [76–78]. In agreement, here we observed a positive correlation between* STAT1* and* IRF1.*

A positive correlation was found between* JAK1 *and* STAT3*, as well as between* JAK1* and* STAT1. *This finding was expected, since these molecules belong to the JAK/STAT signaling pathway [41]. As mentioned, patients without dyslipidemia and diabetes have an increased expression of IFN-gamma [64–68], which activates the JAK/STAT signaling pathway. In agreement, here we observed a positive correlation between* IFNG *with* STAT1, STAT3* and* JAK1 *(Table 4). Similar to* IFNG,* the expressions of* JAK1 *and* STAT3 *were statistically lower in subjects with dyslipidemia (G1, G2, and G3), and significant negative correlations were found between these genes and lipid parameters. Interestingly, the expression of* IL10RB* differed from that of* IL10*, as well as between* IFNG* and* IFNGR1* and* IFNGR2*. We did not find in the literature potential explanations for this observation, but similar results were observed in a previous study of our group related to patients with Down syndrome and periodontal disease [29].

Dyslipidemia and type 2 diabetes have a dysregulatory effect on immune system cells and on wound healing, and, as a result, they increase the susceptibility to periodontitis and other infections [79]. Despite the presence of chronic periodontitis not appearing to influence the systemic expression of the genes studied, this study showed a correlation between lower expression of* IFNG* and* IP10* and higher expression of* IL10* and* IFNA* with the severity of chronic periodontitis (higher percentage of visible plaque, marginal bleeding, BP, PPD ≥ 6 mm, CAL ≥ 5 mm, and suppuration). These results suggest that the systemic expression of these genes may directly influence the periodontal tissue. Moreover, the glycemic control and lipid parameters could be related to the progression of CP. It is important to emphasize that the simplistic classification of proinflammatory or anti-inflammatory cytokines has been challenged by extensive experimental data. In fact, numerous cytokines have both proinflammatory and anti-inflammatory properties, dependent upon the target cells, the cytokine amount, and the activated signaling pathway [35].

The present study has some limitations, including the lack of longitudinal data-tracking of diseases, and no quantification of proteins, such as IL-10 and IFN-gamma in the plasma of patients. In addition, we did not separate cells of the PBMCs, but we think that the gene expression findings were not influenced by a specific cell type because we assessed the counts of lymphocytes, neutrophils, eosinophils, and monocytes in each patient, and they were statistically similar between groups. Despite the mentioned limitations, they did not affect the confidence of the results found.

## 5. Conclusions

Our study provides evidence that leads to the following conclusions: (i) dyslipidemia seemed to be the primary disease associated mainly with increase of* IL10* and* IFNA* and decrease of* IFNG, IP10, IRF1, JAK1, *and* STAT3 *gene expression; (ii) T2D and CP also participate in gene expression, since glucose and periodontal parameters significantly correlated with the mRNA levels of the investigated immune genes.

These findings provide evidence that inflammatory dysregulation associates with dyslipidemia and demonstrated the complex regulation of the systemic immune response, mainly when dyslipidemia is associated with T2D and CP. Moreover, we observed that dyslipidemic patients also affected by poorly controlled diabetes presented increased severity of periodontitis, collectively negatively affecting health and quality of life.

## Supplementary Material

Detailed information regarding sequences of primers and real-time PCR cycling parameters of investigated target genes were (IL10, IL10RA, IL10RB, JAK1, STAT3, SOCS3, IP10, ICAM1, IFNA, IFNARA, IFNAR2, IFNG, IFNGR1, IFNGR2, STAT1, and IRF1).

## Figures and Tables

**Table 1:** Periodontal parameters of the sample (mean ± SD).

Parameters	Group 1 *n* = 30	Group 2 *n* = 30	Group 3 *n* = 30	Group 4 *n* = 30	Group 5 *n* = 30
Number of teeth	22.3 (±4.2)^a^	21.6 (±4.5)^a^	23.2 (±3.8)^a^	24.3 (±3.1)^a^	27.1 (±1.8)
Percentage of sites with visible plaque	76.5 (±17.4)^a,b^	69.8 (±13.0)^a^	70.1 (±15.7)^a^	60.8 (±17.4)^a^	14.8 (±6.2)
Percentage of sites with marginal bleeding	60.9 (±15.2)^a,b,c^	46.9 (±15.9)^a^	40.4 (±14.8)^a^	41.3 (±12.9)^a^	8.7 (±4.8)
Percentage of sites with bleeding on probing	69.3 (±12.8)^a,b,c,d^	53.9 (±13.8)^a^	53.0 (±13.7)^a^	51.4 (±13.2)^a^	12.7 (±5.6)
Mean of the probing depth (mm)	4.1 (±0.5)^a,b,c,d^	3.7 (±0.6)^a^	3.4 (±0.5)^a^	3.7 (±0.4)^a^	2.1 (±0.2)
Percentage of sites with probing depth ≤ 3 mm	43.3 (±14.8)^a,c,d^	57.0 (±15.0)^a^	61.9 (±14.0)^a^	53.3 (±12.5)^a^	98.8 (±1.5)
Percentage of sites with probing depth = 4-5 mm	31.9 (±11.6)^a,b^	31.0 (±11.0)^a,b^	31.0 (±10.6)^a,b^	41.0 (±10.0)^a^	1.2 (±1.5)
Percentage of sites with probing depth ≥ 6 mm	24.8 (±15.9)^a,b,c^	12.0 (±10.8)^a^	7.0 (±10.6)^a^	5.7 (±5.9)^a^	0.0 (±0.0)
Mean of the attachment loss (mm)	4.4 (±0.7)^a,b,c,d^	3.9 (±0.7)^a^	3.6 (±0.5)^a^	3.8 (±0.4)^a^	2.2 (±0.2)
Percentage of sites with attachment loss ≤ 2 mm	13.1 (±8.8)^a^	16.5 (±15.1)^a^	24.8 (±16.2)^a,b^	10.3 (±10.0)^a^	64.5 (±13.9)
Percentage of sites with attachment loss = 3-4 mm	39.8 (±15.3)^b,c^	48.8 (±14.2)^a,b^	51.6 (±10.7)^a^	61.3 (±10.0)^a,c^	35.5 (±13.9)
Percentage of sites with attachment loss ≥ 5 mm	47.1 (±16.2)^a,b,c,d^	34.7 (±17.6)^a^	23.8 (±14.0)^a^	28.4 (±10.5)^a^	0.0 (±0.0)
Number of sites with suppuration	6.8 (±7.0)^a,b,c,d^	4.0 (±3.3)^a^	2.0 (±3.0)	2.0 (±3.7)	0.0 (±0.0)

^a^Significant *P* value in relation to group 5; ^b^significant *P* value in relation to group 4; ^c^significant *P* value in relation to group 3; ^d^significant *P* value in relation to group 2. Comparisons between all groups for nonnormally distributed variables were performed by the Kruskal–Wallis test, and Dunn test was used to correct for multiple comparisons. For normally distributed variables, one-way analysis of variance (ANOVA) test with Holm–Sidak multiple comparison test was used.

**Table 2:** Characteristics of the sample: demographic, physical, biochemical, and diabetic data (mean ± SD).

	Group 1 *n* = 30	Group 2 *n* = 30	Group 3 *n* = 30	Group 4 *n* = 30	Group 5 *n* = 30
Gender (F/M)	18/12	20/10	17/13	19/11	18/12
Age (mean SD)	48.0 (±7.6)^a^	50.3 (±6.7)^a^	49.0 (±7.5)^a^	45.9 (±5.9)^a^	39.3 (±3.6)
BMI (m/Kg^2^)	30.5 (±5.2)^a^	31.4 (±4.1)^a,b^	28.4 (±3.8)	27.3 (±6.4)	24.5 (±3.5)
Waist-to-hip ratio (cm)	1.0 (±0.1)^a,b^	1.0 (±0.1)^a,b^	0.9 (±0.1)^a^	0.9 (±0.1)^a^	0.8 (±0.07)
Abdominal circumference (cm)	104.3 (±14.6)^a^	109.3 (±10.8)^a,b,c^	98.1 (±9.9)	98.2 (±16.9)	87.5 (±10.6)
Fasting glucose (mg/dl)	226.6 (±74.2)^a,b,c,d^	137.5 (±41.4)^a,b,c^	90.0 (±6.4)	90.8 (±7.3)	85.9 (±6.5)
HbA_1c_ (%)	10.4 (±1.9)^a,b,c,d^	6.6 (±0.9)^a,b,c^	5.4 (±0.6)	5.1 (±0.6)	5.4 (±0.21)
Insulin (U/L)	19.7 (±20.9)^a^	21.1 (±21.5)^a,b^	12.6 (±8.5)	11.1 (±12.7)	7.1 (±4.3)
HOMA	12.7 (±15.9)^a,b,c^	6.8 (±5.2)^a,b,c^	2.6 (±1.8)	2.9 (±3.5)	1.6 (±1.0)
Total cholesterol (mg/dl)	242.7 (±37.8)^a,b^	243.4 (±42.9)^a,b^	246.1 (±42.3)^a,b^	171.6 (±18.5)	180.3 (±21.5)
HDL cholesterol (mg/dl)	44.8 (±9.5)	46.1 (±10.5)	50.7 (±11.1)	48.4 (±12.6)	49.3 (±10.1)
LDL cholesterol (mg/dl)	153.4 (±37.0)^a,b^	147.3 (±44.3)^a,b^	156.4 (±44.1)^a,b^	103.8 (±17.4)	113.5 (±18.1)
Triglycerides (mg/dl)	216.9 (±94.6)^a,b^	249.8 (±104.1)^a,b^	194.1 (±80.6)^a,b^	93.9 (±35.9)	87.4 (±27.6)

^a^Significant *P* value in relation to group 5; ^b^significant *P* value in relation to group 4; ^c^significant *P* value in relation to group 3; ^d^significant *P* value in relation to group 2. Comparisons between all groups for nonnormally distributed variables were performed by the Kruskal–Wallis test, and Dunn test was used to correct for multiple comparisons. For normally distributed variables, one-way analysis of variance (ANOVA) test with Holm–Sidak multiple comparison test was used.

**Table 3:** Adjusted correlation between gene expression and physical parameters and glycemic and lipid profiles.

	Physical parameters			Glycemic and lipid profiles					
	BMI (m/kg^2^)	Waist-to-hip ratio (cm)	Waist circumference (cm)	Fasting glucose (mg/dl)	HbA1c (%)	HOMA	Total cholesterol (mg/dl)	LDL cholesterol (mg/dl)	Triglycerides (mg/dl)
*IL10*	*0.31*	*0.37*	*0.30*	*0.38*	*0.37*	0.15	*0.57*	*0.45*	*0.42*
*IFNA*	*0.22*	*0.23*	0.13	*0.55*	*0.65*	*0.33*	*0.39*	*0.30*	*0.34*
*IFNG*	**−0.23**	**−0.38**	**−0.22**	**−0.48**	**−0.44**	−0.14	**−0.54**	**−0.40**	**−0.48**
*IRF1*	**−0.26**	**−0.26**	**−0.18**	**−0.28**	**−0.37**	**−0.19**	**−0.49**	**−0.38**	**−0.39**
*SOCS3*	*0.21*	*0.21*	*0.26*	*0.26*	*0.28*	*0.23*	*0.29*	*0.26*	*0.20*
*IP10*	**−0.23**	**−0.30**	**−0.24**	**−0.39**	**−0.33**	−0.14	**−0.50**	**−0.38**	**−0.55**
*JAK1*	**−0.29**	**−0.22**	**−0.20**	**−0.26**	**−0.23**	−0.15	**−0.44**	**−0.32**	**−0.37**
*STAT1*	**−0.19**	**−0.18**	−0.14	**−**0.01	**−**0.01	0.07	**−**0.06	0.03	**−0.33**
*STAT3*	−0.16	−0.17	−0.05	**−0.24**	**−0.22**	−0.06	**−0.38**	**−0.27**	**−0.34**

Spearman's correlation coefficients are shown (*r*; *α* = 5%) adjusted for age and gender. Significant correlations (*P* < 0.05) are marked: italic means positive significant correlation and bold means negative significant correlation.

**Table 4:** Adjusted correlation of expressions between the investigated genes.

	*IL10*	*IL10RA*	*IL10RB*	*JAK1*	*STAT1*	*STAT3*	*ICAM1*	*IRF1*	*SOCS3*	*IP10*	*IFNG*	*IFNGR1*	*IFNGR2*	*IFNA*	*IFNAR1*	*IFNAR2*
*IL10*	1.00															
*IL10RA*	−0.15	1.00														
*IL10RB*	−**0.56**	*0.23*	1.00													
*JAK1*	−**0.59**	*0.44*	*0.63*	1.00												
*STAT1*	−0.13	*0.33*	*0.50*	*0.39*	1.00											
*STAT3*	−**0.50**	*0.29*	*0.59*	*0.61*	*0.46*	1.00										
*ICAM1*	0.08	*0.38*	*0.20*	0.08	*0.48*	*0.39*	1.00									
*IRF1*	−**0.57**	*0.31*	*0.47*	*0.57*	*0.28*	*0.62*	*0.23*	1.00								
*SOCS3*	*0.35*	*0.31*	−0.06	−0.17	*0.23*	−0.02	*0.34*	−0.13	1.00							
*IP10*	−**0.67**	0.13	*0.64*	*0.62*	*0.37*	*0.48*	0.04	*0.49*	−**0.22**	1.00						
*IFNG*	−**0.75**	0.07	*0.57*	*0.56*	*0.20*	*0.52*	−0.03	*0.51*	−**0.26**	*0.72*	1.00					
*IFNGR1*	*0.64*	0.01	−**0.33**	−**0.43**	0.03	−**0.36**	0.15	−**0.53**	*0.51*	−**0.44**	−**0.59**	1.00				
*IFNGR2*	*0.44*	−0.10	−0.15	−**0.32**	0.05	−**0.24**	0.10	−**0.43**	*0.33*	−**0.29**	−**0.37**	*0.65*	1.00			
*IFNA*	*0.49*	0.06	−**0.37**	−**0.43**	−0.02	−**0.35**	0.13	−**0.48**	*0.36*	−**0.45**	−**0.57**	*0.48*	*0.45*	1.00		
*IFNAR1*	*0.38*	0.13	−0.14	−0.07	0.09	−0.14	*0.20*	−**0.39**	*0.39*	−**0.21**	−**0.35**	*0.67*	*0.48*	*0.36*	1.00	
*IFNAR2*	*0.18*	0.17	−0.07	−0.13	0.08	−0.09	*0.23*	−**0.24**	*0.41*	−0.16	−**0.23**	*0.47*	*0.48*	*0.40*	*0.52*	1.00

Spearman's correlation coefficients are shown (*r*; *α* = 5%) adjusted for age and gender. Significant correlations (*P* < 0.05) are marked: italic means positive significant correlation and bold means negative significant correlation.

**Table 5:** Adjusted correlation between gene expression and periodontal parameters.

	Visible plaque (% sites)	Marginal bleeding (% sites)	BP (% sites)	PPD ≤ 3 mm (% sites)	PPD 4-5 mm (% sites)	PPD ≥ 6 mm (% sites)	CAL ≤ 2 mm (% sites)	CAL 3-4 mm (% sites)	CAL ≥ 5 mm (% sites)	Suppuration (No sites)
*IL10*	*0.37*	*0.37*	*0.40*	−**0.28**	0.16	*0.29*	−**0.23**	−0.08	*0.33*	*0.19*
*IFNA*	*0.34*	*0.37*	*0.36*	−**0.27**	−0.06	*0.39*	−**0.19**	−0.15	*0.35*	*0.39*
*IFNG*	−**0.45**	−**0.40**	−**0.45**	*0.35*	−**0.20**	−**0.39**	*0.30*	0.05	−**0.38**	−**0.22**
*IP10*	−**0.27**	−**0.26**	−**0.28**	*0.22*	−0.07	−**0.31**	0.09	*0.21*	−**0.27**	−0.11
*IRF1*	−**0.30**	−**0.31**	−**0.30**	0.16	−0.01	−**0.27**	0.07	*0.22*	−**0.25**	−0.13
*SOCS3*	*0.18*	*0.19*	0.12	−0.12	−0.13	*0.35*	−0.06	−0.19	*0.23*	*0.32*

Spearman's correlation coefficients are shown (*r*; *α* = 5%) adjusted for age and gender. Significant correlations (*P* < 0.05) are marked: italic means positive significant correlation and bold means negative significant correlation. BP: bleeding on probing; PPD: periodontal pocket depth; CAL: clinical attachment loss.
