# Supplementary material for: Dyslipidemia rather than Type 2 Diabetes Mellitus or Chronic Periodontitis Affects the Systemic Expression of Pro- and Anti-Inflammatory Genes
Source: Mediators Inflamm. 2017 Feb 20;2017:1491405. doi: 10.1155/2017/1491405 (PMC5337859; doi:10.1155/2017/1491405)
Supplement: Supplementary file 1 — Detailed information regarding sequences of primers and real-time PCR cycling parameters of investigated target genes were (IL10, IL10RA, IL10RB, JAK1, STAT3, SOCS3, IP10, ICAM1, IFNA, IFNARA, IFNAR2, IFNG, IFNGR1, IFNGR2, STAT1, and IRF1). [file 1491405.f1.docx]

| **Supplemental Table 1.** Primer Information: GenBank accession number of deposited mRNA sequences of the investigated genes, primer sequences and amplicon sizes. | | | | | |  |
| --- | --- | --- | --- | --- | --- | --- |
| Gene | Accession Number | Primer | | Amplicon Size | |  |
|  |  | Sense and Antisense Sequences | |  |  |  |
| *GAPDH* | NM_ 001256799.2 | F | 5’ CAAGGCTGTGGGCAAGGT 3’ | | 88 | |
|  |  | R | 5’ CAGGTCAGGTCCACCACTGA 3’ | |  |  |
| *IL10* | NM_000572 | F | 5’ GCCAGGGCACCCAGTCT 3’ | | 74 | |
|  |  | R | 5’ TCGGAGA TCTCGA AGCA TGTT 3’ | |  |  |
| *IL10RA* | NM_001558 | F | 5’ CGCTCCTGAGGTATGGAATAGAG 3’ | | 115 | |
|  |  | R | 5’ GCCCGGT AGCCA TTGCT 3’ | |  |  |
| *IL10RB* | NM_000628 | F | 5’ AA TGGAGTGAGCCTGTCTGTGA 3’ | | 90 | |
|  |  | R | 5’ TGAAGACCGAGGCCA TGAG 3’ | |  |  |
| *JAK1* | NM_002227 | F | 5’ GTCACAACCTCTTTGCCCTGTAT 3’ | | 91 | |
|  |  | R | 5’ CGGAGGGACA TCTTGTCA TCA 3’ | |  |  |
| *STAT3* | NM_139276 | F | 5’ TGCTGAAATCATCATGGGCTATA 3’ | | 90 | |
|  |  | R | 5’ TCCTTGGGAATGTCAGGATAGAG 3’ | |  |  |
| *SOCS3* | NM_003955 | F | 5’ TCCCCCCAGAAGAGCCTATT 3’ | | 90 | |
|  |  | R | 5’ GTCTTCCGACAGAGA TGCTGAA 3’ | |  |  |
| *IP10* | NM_001504 | F | 5’ ACTGCCCTTCTCA TTTGGAAAC | | 100 | |
|  |  | R | 5’ CCTGGGCTGTGGCTTCA T 3’ | |  |  |
| *ICAM1* | NM_000201 | F | 5’ GAAAA TTCCCAGCAGACTCCAA 3’ | | 94 | |
|  |  | R | 5’ CGA TGGGCAGTGGGAAAGT 3’ | |  |  |
| *IFNG* | NM_000619 | F | 5’ GAAACGAGATGACTTCGAAAAGC 3’ | | 113 | |
|  |  | R | 5’ GCTGCTGGCGACAGTTCA 3’ | |  |  |
| *IFNGR1* | NM_000416 | F | 5’ GGTCTGTGAAGAGCCGTTGTC 3’ | | 142 | |
|  |  | R | 5’ CGGGACCACGTCAGGAATAT 3’ | |  |  |
| *IFNGR2* | NM_005534 | F | 5’ GGAAAAGGAGCAAGAAGATGTTCT 3’ | | 93 | |
|  |  | R | 5’ AGCTCCGATGGCTTGATCTC 3’ | |  |  |
| *IFNA* | NM_002169 | F | 5’ GAAGAATCTCTCCTTTCTCCTGCC 3’ | | 110 | |
|  |  | R | 5’ ATGGAGGACAGAGATGGCTTG 3’ | |  |  |
| *IFNAR1* | NM_000629 | F | 5’ CACTTCTTCATGGTATGAGGTTGACT 3’ | | 96 | |
|  |  | R | 5’ ATTGCCTTATCTTCAGCTTCTAAATGT 3’ | |  |  |
| *IFNAR2* | NM_207584 | F | 5’ TCATATGATTCGCCTGATTACACA 3’ | | 108 | |
|  |  | R | 5’ TGGTACAATGGAGTGGTTTTTTAATT 3’ | |  |  |
| *STAT1* | NM_139266 | F | 5’ GTGTTATGGGACCGCACCTT 3’ | | 107 | |
|  |  | R | 5’ AAGACCAGCGGCCTCTGA 3’ | |  |  |
| *IRF1* | NM_002198 | F | 5’ GCATGGCTGGGACATCAAC 3’ | | 97 | |
|  |  | R | 5’ CTTGGGATCTGGCTCCTTTTC 3’ | |  |  |
| *F = Primer forward; R = Primer reverse.* | | | | | |  |
